# Glucocorticoid Receptor (*NR3C1*) Variants Associate with the Muscle Strength and Size Response to Resistance Training

**DOI:** 10.1371/journal.pone.0148112

**Published:** 2016-01-28

**Authors:** Garrett I. Ash, Matthew A. Kostek, Harold Lee, Theodore J. Angelopoulos, Priscilla M. Clarkson, Paul M. Gordon, Niall M. Moyna, Paul S. Visich, Robert F. Zoeller, Thomas B. Price, Joseph M. Devaney, Heather Gordish-Dressman, Paul D. Thompson, Eric P. Hoffman, Linda S. Pescatello

**Affiliations:** 1 Department of Kinesiology and Human Performance Laboratory, University of Connecticut, Storrs, Connecticut, United States of America; 2 Department of Behavioral and Social Sciences, Brown University School of Public Health, Providence, Rhode Island, United States of America; 3 School of Health Sciences, Emory & Henry College, Emory, VA, United States of America; 4 Department of Kinesiology, University of Massachusetts, Amherst, Massachusetts, United States of America; 5 Department of Health, Human Performance and Recreation, Baylor University, Waco, Texas, United States of America; 6 School of Health and Human Performance, Dublin City University, Dublin, Ireland; 7 Exercise and Sport Performance Department, University of New England, Biddeford, Maine, United States of America; 8 Department of Exercise Science and Health Promotion, Florida Atlantic University, Boca Raton, Florida, United States of America; 9 Department of Health Sciences, University of Bridgeport, Bridgeport, Connecticut, United States of America; 10 Center for Genetic Medicine Research, Children's National Medical Center, Washington, District of Columbia, United States of America; 11 Division of Cardiology, Henry Low Heart Center, Hartford Hospital, Hartford, Connecticut, United States of America; 12 Institute for Systems Genomics, University of Connecticut, Storrs, Connecticut, United States of America; Virginia Commonwealth University, UNITED STATES

## Abstract

Glucocorticoid receptor (*NR3C1)* polymorphisms associate with obesity, muscle strength, and cortisol sensitivity. We examined associations among four *NR3C1* polymorphisms and the muscle response to resistance training (RT). European-American adults (n = 602, 23.8±0.4yr) completed a 12 week unilateral arm RT program. Maximum voluntary contraction (MVC) assessed isometric strength (kg) and MRI assessed biceps size (cm^2^) pre- and post-resistance training. Subjects were genotyped for *NR3C1* -2722G>A, -1887G>A, -1017T>C, and +363A>G. Men carrying the -2722G allele gained less relative MVC (17.3±1.2vs33.5±6.1%) (p = 0.010) than AA homozygotes; men with -1887GG gained greater relative MVC than A allele carriers (19.6±1.4vs13.2±2.3%) (p = 0.016). Women carrying the -1017T allele gained greater relative size (18.7±0.5vs16.1±0.9%) (p = 0.016) than CC homozygotes. We found sex-specific *NR3C1* associations with the muscle strength and size response to RT. Future studies should investigate whether these associations are partially explained by cortisol’s actions in muscle tissue as they interact with sex differences in cortisol production.

## Introduction

The glucocorticoid receptor (*NR3C1*) gene on chromosome 5q31-32 codes for the NR3C1 protein that binds glucocorticoid hormones within the liver, muscle, and vasculature influencing metabolism and cardiovascular function [1–3]. *NR3C1* genetic variants may influence these physiological systems and responses by altering NR3C1 protein function [1,2]. For example, humans carrying the G allele of the *NR3C1* +363 A>G (Asn>Ser; rs56149945, formerly rs6195) single nucleotide polymorphism (SNP) on exon 2 of *NR3C1* bind the glucocorticoid hormone cortisol at a more rapid rate than those with the AA genotype [4]. *NR3C1* +363 G allele carriers have a greater body mass index (BMI) [4,5], total cholesterol to high density lipoprotein ratio [6], and triglyceride levels [6] as well as an increased risk of coronary artery disease [6], and diabetes mellitus [4,7]than those with the AA genotype. However, other investigators have not found *NR3C1* +363 A>G genotype differences with body composition [8,9], blood pressure [5], insulin resistance [5], and cardiovascular disease [10].

In addition, the *NR3C1* 9β A>G (rs6198), *NR3C1 Bcl*I C>G (rs41423247), and *NR3C1* Tth111I (rs10052957) SNPs and the *NR3C1* ER22/23EK (Arg>Lys; rs6189/6190) haplotype are associated with cortisol uptake [11–13], NR3C1 isoform abundance [14], inflammation [10], cardiovascular disease [10] and isometric and dynamic muscle strength [15]. van Rossum et al. [15] investigated the influence of the *NR3C1* ER22/23EK haplotype on isometric and dynamic muscle strength, body composition, and habitual physical activity among 337 healthy, adult men and women 32-36yr. *NR3C1* ER22/23EK associated with isometric and dynamic muscle strength, lean body mass, and thigh circumference among men; whereas *NR3C1* ER22/23EK tended to associate with waist and hip circumference among women, but these associations did not achieve statistical significance.

*NR3C1* ER22/23EK associates with cortisol uptake [12]. Therefore, van Rossum et al. [15] speculated this variant may alter NR3C1 protein function, subsequently influencing cortisol binding and uptake and cortisol-stimulated muscle catabolism, and ultimately, muscle strength and size. In support of the findings of van Rossum et al. [15], Peeters and colleagues [16] reported adults with the *NR3C1* ER22/ER22 genotype exhibited an inverse correlation among serum cortisol levels and muscle strength and size, but no such associations existed among carriers of the ER23 allele.

We have found genetic factors account for a small amount of variability in the muscle size and strength response to 12 wk of upper body, unilateral resistance training (RT) among a large sample of young, healthy women and men in the Functional Single Nucleotide Polymorphisms Associated with Muscle Size and Strength study (FAMuSS) [17–20]. These associations were frequently modified by sex [20]. In the present study, we hypothesized *NR3C1* +363 G allele carriers among the FAMuSS cohort would exhibit less muscle strength and size at baseline and gain less muscle strength and size in response to RT than non-carriers; since they bind cortisol more rapidly than non-carriers [4], they may exhibit greater cortisol-stimulated muscle catabolism and therefore lesser muscle strength and size before and after RT. We also hypothesized -2722 G>A (rs10482614), -1887 G>A (rs10482616), and -1017 T>C (rs4634384) in the 5’ untranslated region of *NR3C1* that are implicated in transcriptional regulation [14] would associate with muscle size and strength at baseline and in response to RT among the FAMuSS cohort; and as van Rossum et al. [15] found, these associations would be sex dependent.

## Methods

FAMuSS methods and overall results have been described in detail elsewhere [17,20–22]. The Institutional Review Board of each site (University of Connecticut, Dublin City University, University of Massachusetts, Central Michigan University, University of Central Florida, Florida Atlantic University, West Virginia University, Hartford Hospital, Children’s National Medical Center, and Yale University) approved the protocol, and all subjects gave written informed consent to participate. Briefly, volunteers were recruited to complete a 12 wk progressive, unilateral RT program to improve the strength and size of elbow flexor and extensor muscles in the non-dominant arm. Isometric muscle strength was measured as biceps maximum voluntary contraction (MVC), dynamic muscle strength as one repetition maximum (1RM) for a standard preacher curl exercise, and muscle size as magnetic resonance imaging (MRI) of biceps cross-sectional area (CSA). Prior to RT subjects provided a blood sample from which DNA was extracted. All data are available as a supplemental file (**S1 File**).

### Setting

The sites for recruiting, RT, and muscle testing included the University of Connecticut, Dublin City University, University of Massachusetts, Central Michigan University, University of Central Florida, Florida Atlantic University, West Virginia University, and Hartford Hospital. All genotyping was performed at Children’s National Medical Center and MRI image analysis was conducted at Yale University.

### Inclusion Criteria

Subjects were excluded if they took corticosteroids, anabolic steroids, antihypertensive or antilipidemic medications, diuretics, Depo-Provera contraceptive injection, Clenbuterol, Rhinocort nasal inhaler, lithium, or nonsteroidal anti-inflammatory medications. They were also excluded if they: took dietary supplements to enhance muscle size and strength or weight; had chronic medical conditions; had metal implants in the arms, eyes, head, brain, neck, or heart; consumed >2 alcoholic drinks/day; performed RT or other physical activity involving repetitive arm use within the past year; and/or were seeking to gain or lose weight or had a weight change of >5 lb in the past 3 months. Furthermore, subjects were instructed not to alter their dietary habits, habitual physical activity behavior, or otherwise gain or lose weight during the study. Upon enrollment we measured body weight and height to calculate BMI, and subjects completed the Paffenbarger Physical Activity Questionnaire [23] to assess habitual physical activity. Adherence to usual diet was monitored by repeated weight measurements throughout the study. We compensated subjects $100-$150 depending on site for their time and effort.

### Isometric and Dynamic Strength

We assessed isometric strength as MVC in the trained (non-dominant) elbow flexor muscles with a preacher curl bench and strain gauge (model 32628CTL, Lafayette Instrument Company, Lafayette, IN) [17,20–22]. Briefly, subjects were instructed to sustain maximal effort for 3 s and repeat 3 times with 1 min between contractions. If any contraction differed by more than 5 lb (2.2 kg) from the other two contractions, subjects were informed of this and asked to perform up to two more contractions until values were consistent. Maximal force was recorded in kg. Baseline MVC was determined as the average of two pre-RT assessments following a familiarization assessment, and post-RT MVC was determined as the average of two post-RT assessments immediately before and 48hr after the last training session. We also assessed dynamic strength of the same arm by determining 1RM, or the maximum weight the subject could successfully lift for one repetition of the preacher curl exercise. We tested 1RM once pre- and post-RT, after the final MVC test on both occasions so that muscle fatigue from 1RM testing did not influence MVC results.

### Muscle Size

We used 1.5T MRI to measure CSA of the trained (non-dominant) biceps brachii [17,20–22]. Measurements were taken at the arm’s maximum circumference (point of measure; POM) prior to RT to determine baseline CSA and within 96hr of the last RT session to determine post-RT CSA. All measurements were performed by a trained MRI technician. Briefly, the POM was located by the subject performing a maximal biceps contraction with the shoulder abducted 90°, elbow flexed 90°, and hand open. The POM was marked with a radiographic bead (Beekley Spots, Beekley Corp., Bristol, CT). MRI took 15 spoiled gradient axial slice images, perpendicular to the long axis of the humerus with the 8^th^ and 9^th^ slices aligned with the POM. Slice thickness was 16 mm, interslice gap was 0 mm, time to echo was 1.9 s, time to repeat was 200 ms, flip angle was 30°, and flow artifact suppression was utilized. Imaging was proximal to distal, utilizing 256x192 matrix resolution and 22x22 cm field of view. Matlab (The Math Works, Inc., Natick, MA) was used to trace the muscle region, quantify its size in pixels, and convert this size to cm^2^ based on matrix resolution and field of view. Radiographic bead size was assessed to check standardization between testing sites.

### Resistance Training Protocol

RT was performed unilaterally in the non-dominant arm. Subjects attended supervised RT sessions twice weekly at least 48 hr apart for 12 wk. The program was designed primarily to train the elbow flexors and secondarily to train the elbow extensors for balance. Exercises included biceps preacher curl, biceps concentration curl, standing biceps curl, overhead triceps, and triceps kickback. Exercises were performed using dumbbells (Powerblocks, Intellbell, Inc., Owatonna, MN) and incorporated the preacher curl bench as needed. At the start of RT, subjects performed 3 sets of 12 repetitions at 65–75% of 1RM. At week 5 sets were reduced to 8 repetitions at a greater intensity of 75–82% 1RM and at week 10 to 6 repetitions at 83–90% 1RM. Subjects took 2 s for the concentric and 2 s for the eccentric phase of each repetition. Recovery between sets was 2 min.

### Genotyping

Blood was drawn into vacutainer tubes containing ethylenediamine teraacetic acid. These tubes were sent to Children’s National Medical Center where DNA was extracted using Puregene kits (Gentra Systems, Inc., Minneapolis, MN). An MWG robot (MWG Biotech, Ebersberg, Germany) performed genotyping using TaqMan^®^ allele discrimination assays. These assays utilized Taq polymerase oligonucleotide probes that were labeled with a separate fluorophore. For each SNP an allele-specific polymerase chain reaction (PCR) was completed. PCR mixtures included 10 ng genomic DNA, 900 nM forward and reverse PCR primers, 200 nM each of two allele specific probes, and TaqMan^®^ Universal PCR Master Mix, No AmpErase^®^ UNG (Applied Biosystems, Foster City, CA) to form a final volume 10 μl. This mixture was kept at 95–C for 10 min for denaturation, followed by 44 cycles of 15 s at 92–C alternated with 1 min at 60–C for annealing. A customized 7900HT system (Life Technologies, Carlsbad, CA) was used to detect fluorophore ratios, and thereby call alleles. Data were processed using SDS v2.3 software. All gels were called by two investigators, and if any disagreement in genotyping was found, the genotyping was repeated. Subjects were genotyped for *NR3C1*–2722 G>A, *NR3C1*–1887 G>A, *NR3C1–*1017 T>C, and *NR3C1* +363 A>G with ≥98% success.

FAMuSS in total enrolled 949 European-American subjects from which 680 were genotyped for the four *NR3C1* SNPs we examined in this sub-study. From this subsample of 680 participants, the RT program was completed by 602 participants and were genotyped for *NR3C1*–2722 G>A (GG: n = 420, GA: n = 153, AA: n = 20), *NR3C1*–1887 G>A (GG: n = 436, GA: n = 152, AA: n = 12), *NR3C1–*1017 T>C (TT: n = 156, TC: n = 300, CC: n = 139), and *NR3C1* +363 A>G (AA: n = 562, AG: n = 30, GG: n = 7).

### Statistical Analysis

We determined descriptive statistics for all study variables and by genotype groups. We verified all SNPs were in Hardy-Weinberg Equilibrium with the chi-square test for the total sample (p>0.05). Chi-square tests also determined no *NR3C1* SNPs were in linkage disequilibrium (r^2^<0.20). RT dependent variables included baseline muscle strength (MVC) and size (CSA) and the change in relative (post-RT–pre-RT/pre-RT × 100) muscle strength (MVC) and size (CSA) before versus after RT. ANCOVA tested associations among *NR3C1* genotypes and the muscle strength and size phenotypes with sex as a fixed factor and covariates including age BMI, and pre-intervention physical activity index (kcal/wk), the sum of energy expended in walking, stair climbing, and participation in sports and recreational activities.

Significant genetic variants from the main ANCOVA models were subjected to post hoc pairwise comparisons among the 3 genotypes of each variant. If the heterozygous genotype did not differ significantly from one of the homozygous genotypes, it was combined with that homozygous genotype to create a dominant or recessive model for each phenotype examined. Thus, we utilized a recessive model for *NR3C1*–2722 G>A (i.e., GG/GA vs AA), dominant for *NR3C1*–1887 G>A (i.e., GG vs GA/AA), recessive for *NR3C1–*1017 T>C (i.e., TT/TC vs CC), and dominant for *NR3C1* +363 A>G (i.e., AA vs AG/GG). Pairwise comparisons were then repeated. P-values were adjusted for multiple comparisons by the Sidak method. Because of significant genotype*sex interactions for all phenotypes examined, data were analyzed and presented separately by sex. Proportion of variance in phenotype due to *NR3C1* genotype was tested using the likelihood ratio between the full regression model including genotype, age, and BMI and the constrained model including only age and BMI. Significance was p≤0.05. All analyses were performed using SPSS 14.0 for Windows except for the significance of likelihood ratios that were determined using SAS 9.1.3 for Windows. We report results for MVC and CSA but not 1RM, since 1RM baseline and change following RT did not associate with any *NR3C1* genotypes among men or women (p>0.05).

## Results

### Subject Characteristics

Healthy adults (232 men and 370 women) of European-American descent comprised the cohort. Age (men, 24.1±0.4 vs women, 23.5±0.3 yr) and BMI (men 25.2±0.3 vs women 24.0±0.2 kg*m^-2^) did not differ by sex (p>0.05).

### *NR3C1* Genetic Variants and Muscle Strength and Size

#### NR3C1–2722 G>A

**Fig 1:**
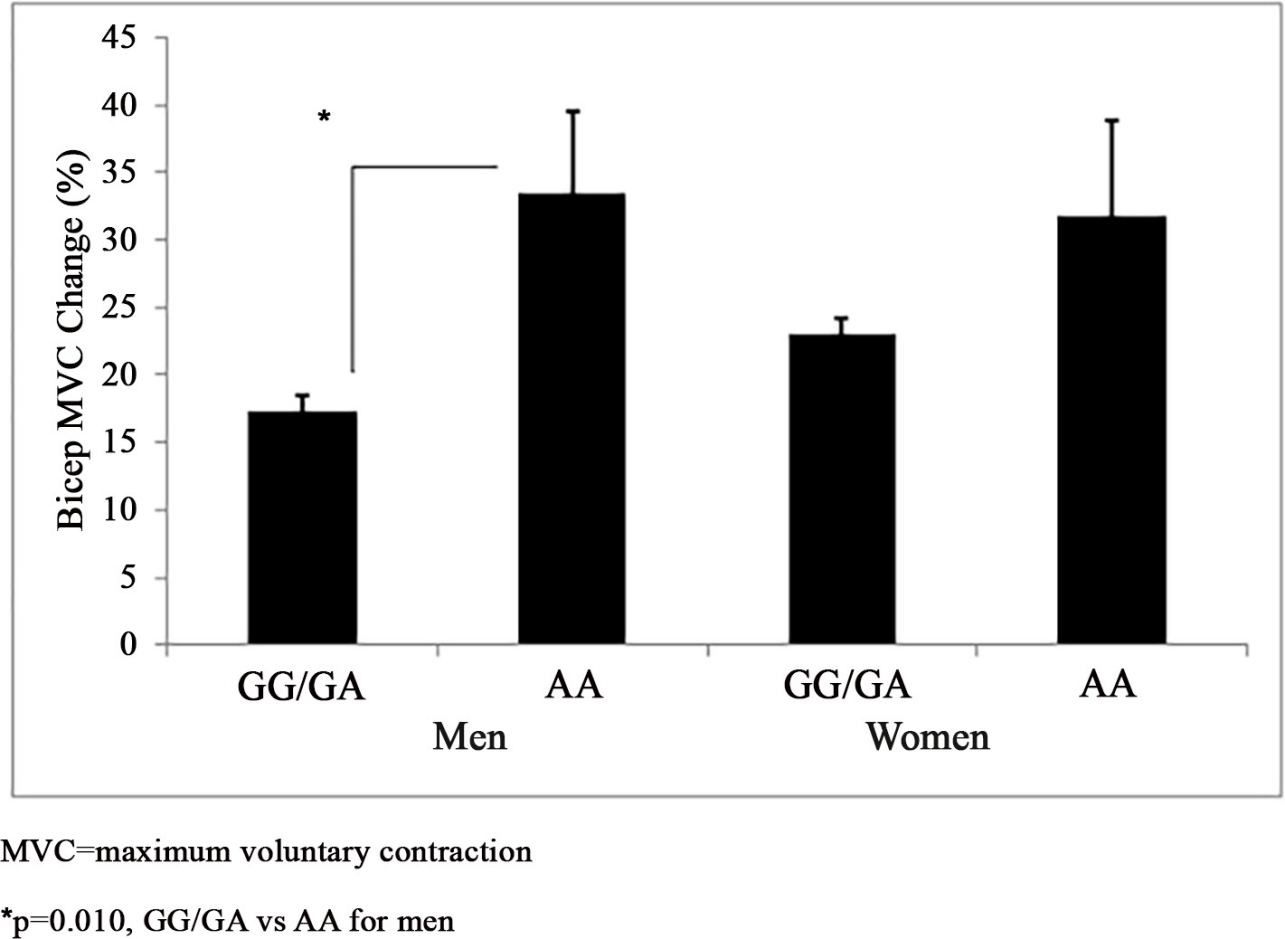
*NR3C1*–2722 G>A and Muscle Strength Response to Resistance Training. *NR3C1*–2722 G>A (rs10482614) genotypes grouped by a recessive model and their association with the relative muscle strength response to resistance training by sex adjusted for age (mean±SEM).

*Muscle Strength*: *NR3C1*–2722 G>A genotype did not associate with baseline isometric muscle strength among men (GG/GA n = 207, 63.4±1.3 kg vs AA n = 8, 56.1±6.7 kg) (p>0.05). Men carrying the *NR3C1–*2722 G allele gained less relative MVC than AA homozygotes (17.3±1.2% vs 33.5±6.1%) (p = 0.010) (Fig 1). *NR3C1*–2722 G>A accounted for 3.2% of variance in the relative MVC response among men (p<0.001). *NR3C1–*2722 G>A genotype did not associate with baseline MVC (GG/GA n = 348, 30.7±0.6 kg vs AA n = 11, 32.2±3.5 kg) or the relative change in MVC (23.0±1.2% vs 31.8±7.0%) among women (p>0.05).

*Muscle Size*: *NR3C1*–2722 G>A genotype did not associate with baseline muscle size (GG/GA n = 211, 20.6±0.4 cm^2^ vs AA n = 9, 21.6±1.7 cm^2^) or the relative change in muscle size (20.9±0.7% vs 18.9±3.3%) among men (p>0.05). *NR3C1*–2722 G>A genotype did not associate with baseline muscle size (GG/GA n = 331, 13.3±0.2 cm^2^ vs AA n = 11, 14.0±0.9 cm^2^) or the relative change in muscle size (18.2±0.5% vs 16.9±2.6%) among women (p>0.05).

#### NR3C1–1887 G>A

**Fig 2:**
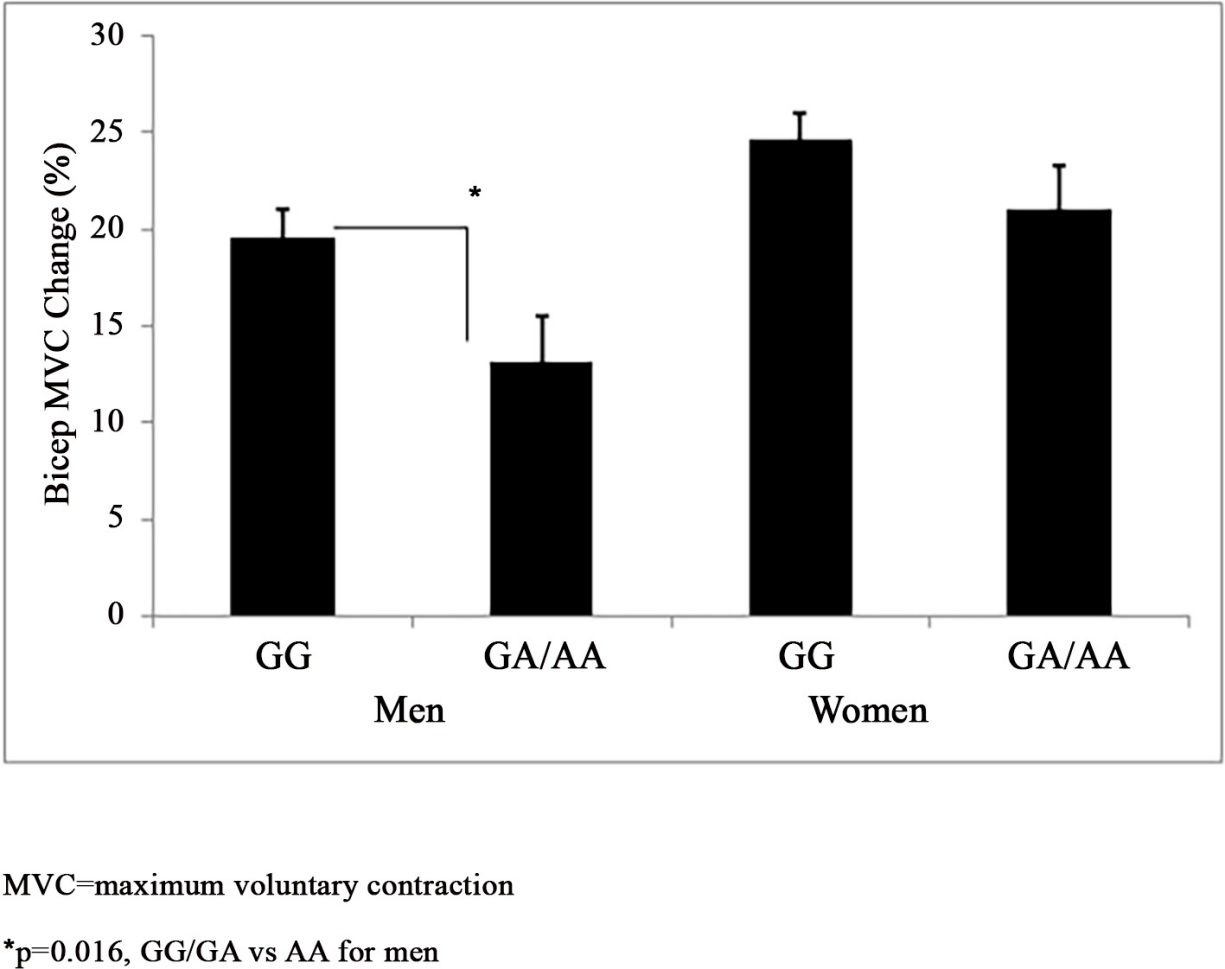
*NR3C1*–1887 G>A and Muscle Strength Response to Resistance Training. *NR3C1*–1887 G>A (rs10482616) genotypes grouped by a dominant model and their association with the relative muscle strength response to resistance training by sex (mean±SEM).

*Muscle Strength*: *NR3C1*–1887 G>A genotype did not associate with baseline MVC among men (GG n = 160, 63.8±2.5 kg vs GA/AA n = 57, 62.9±1.5 kg). Men with the *NR3C1–*1887 GG genotype gained greater relative MVC than A allele carriers (19.6±1.4% vs 13.2±2.3%) (p = 0.016) (Fig 2). *NR3C1*–1887 G>A accounted for 2.4% of the variance in the relative MVC response among men (p<0.001). *NR3C1–*1887 G>A genotype did not associate with baseline MVC (GG n = 260, 30.5±0.7 kg vs GA/AA n = 103, 31.2±1.1 kg) or the relative change in MVC (24.6±1.4% vs 21.2±2.3%) among women (p>0.05).

*Muscle Size*: *NR3C1*–1887 G>A genotype did not associate with baseline muscle size (GG n = 163, 20.8±0.4 cm^2^ vs GA/AA n = 56, 20.1±0.7 cm^2^) or the relative change in muscle size (20.3±0.8% vs 22.5±1.3%) among men (p>0.05). *NR3C1*–1887 G>A genotype did not associate with baseline muscle size (GG n = 250, 13.3±0.2 cm^2^ vs GA/AA n = 96, 13.1±0.3 cm^2^) or the relative change in muscle size (18.0±0.5% vs 18.6±0.9%) among women (p>0.05).

#### NR3C1–1017 T>C

*Muscle Strength*: *NR3C1*–1017 T>C genotype did not associate with baseline MVC (TT/TC n = 166, 62.8±1.5 kg vs CC n = 48, 65.1±2.8 kg) or the relative change in MVC among men (17.4±1.3% vs 18.6±2.5%) (p>0.05). *NR3C1*–1017 T>C genotype did not associate with baseline MVC (TT/TC n = 275, 30.6±0.7 kg vs CC n = 86, 31.5±1.3 kg) or the relative change in MVC (22.3±1.4% vs 26.0±2.5%) among women (p>0.05).

**Fig 3:**
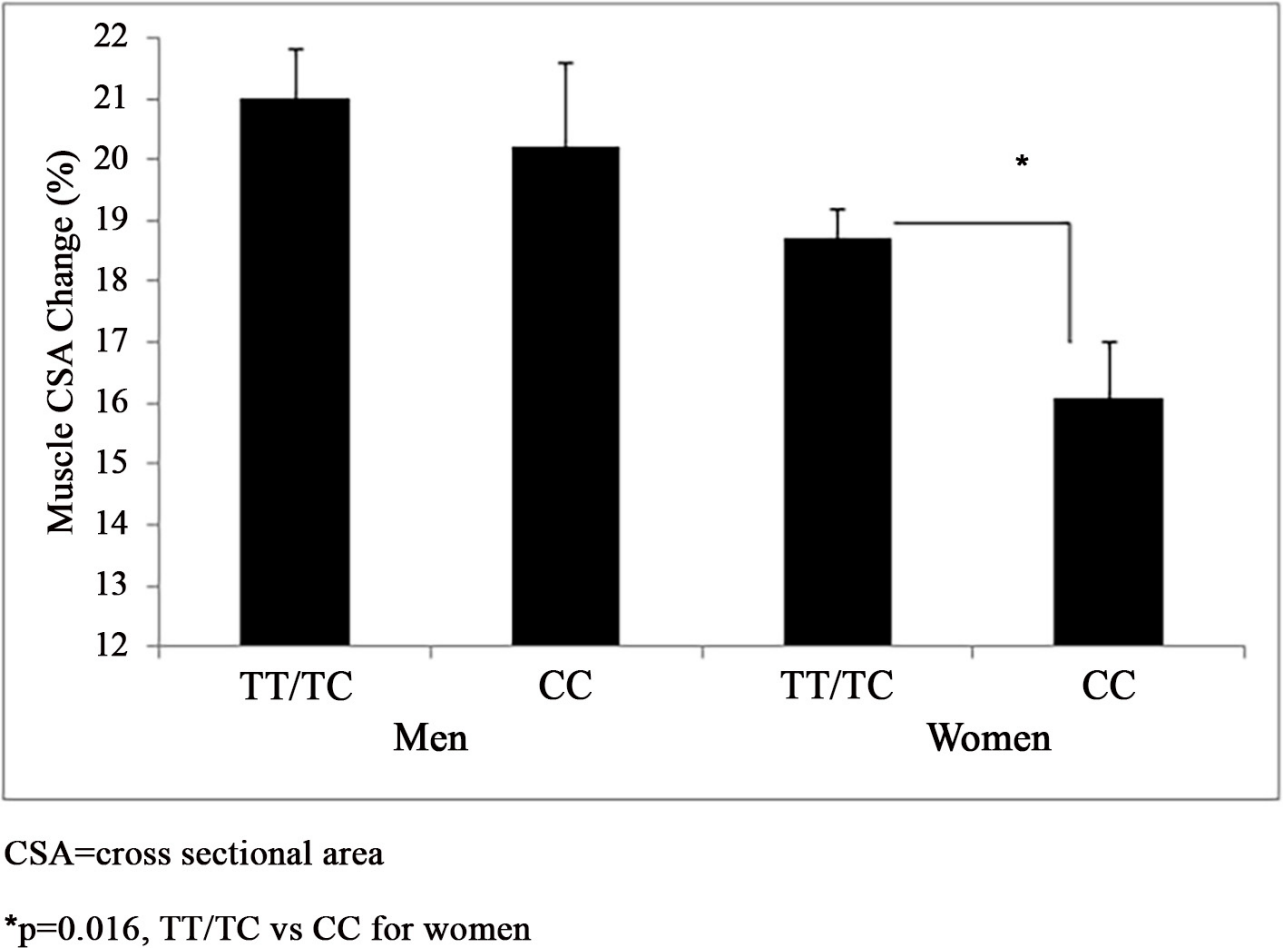
*NR3C1*–1017 T>C and Muscle Size Response to Resistance Training. *NR3C1*–1017 T>C (rs4634384) genotypes grouped by a recessive model and their association with the relative muscle size response to resistance training by sex adjusted for BMI (mean±SEM).

*Muscle Size*: *NR3C1*–1017 G>A genotype did not associate with baseline muscle size (TT/TC n = 170, 20.4±0.4 cm^2^ vs CC n = 51, 21.5±0.7 cm^2^) or the relative change in muscle size (21.1±0.8% vs 20.2±1.4%) among men (p>0.05). *NR3C1*–1017 G>A genotype did not associate with baseline muscle size among women (TT/TC n = 263, 13.2±0.2 cm^2^ vs CC n = 81, 13.4±0.3 cm^2^) (p>0.05). Women carrying the *NR3C1–*1017 T allele gained more relative muscle size than CC homozygotes (18.7±0.5% vs 16.1±0.9%) (p = 0.016) (Fig 3). *NR3C1*–1017 T>C accounted for 1.7% of the variance in the relative muscle size response among women (p<0.001).

#### NR3C1 +363 G>A

*NR3C1* +363 G>A did not associate with baseline muscle strength, size, or the relative change in muscle strength or size among men or women (p>0.05, data not shown).

## Discussion

Evidence supports associations of *NR3C1* genetic variants with human health-related phenotypes, but the findings in the literature are mixed with some studies finding no such associations [2]. Thus, we investigated the influence of four *NR3C1* variants on the muscle strength and size response to RT among the FAMuSS cohort [17], and found sex-specific *NR3C1* genotype associations with the muscle strength and size response to RT. Depending on *NR3C1* genotype the magnitude of the gain in isometric muscle strength in response to RT ranged from 17% to 33% of baseline values among men (Fig 1) and the increase in muscle size as a result of RT ranged from 16% to 19% of baseline values among women (Fig 3). However, we found no associations with *NR3C1–*2722 G>A, *NR3C1*–1887 G>A, *NR3C1*–1017 T>C, and *NR3C1* +363 A>G and baseline muscle strength or size.

The *NR3C1* +363 A>G missense SNP on exon 2 of *NR3C1* is well cited in the literature for associating with a variety of health-related phenotypes [4–7] including cortisol binding [4] and body composition [4,5], which was a primary reason for our examination of its association with muscle phenotypes at baseline and in response to RT in the FAMuSS cohort. However, we found *NR3C1* +363 A>G did not associate with the muscle response to RT. In contrast, the intronic SNPs that we examined (i.e, *NR3C1–*2722 G>A, *NR3C1*-1887 G>A, and *NR3C1–*1017 T>C) in the *NR3C1* 5’ untranslated region did. This 5’ untranslated region contains at least 13 known splice variants of exon 1 which are differentially expressed among bodily tissues [24,25] and individuals [14]. Moreover, it is speculated that the intronic promoter segments among these exon 1 variants are responsible for controlling this differential expression [14,24,25].

In support of this premise, Sinclair et al. [14] found that the *NR3C1* Tth111I intronic variant in the 5’ untranslated region (upstream of *NR3C1* exon 1B) associated with NR3C1-1B mRNA expression among 100 middle-aged adults. Therefore, *NR3C1–*2722 G>A (between exon 1C and exon 1H), *NR3C1*-1887 G>A (between exon 1H and exon 2), and *NR3C1*–1017 T>C (between exon 1H and exon 2) may similarly alter expression of *NR3C1* exon 1 splice variants. Although *NR3C1* exon 1 mRNA is not translated, it still may influence the processing, stability, and encoding of mRNA from exon 2 and other translated regions, ultimately affecting NR3C1 protein abundance, structure, and function [26]. Furthermore, it is via alterations to the NR3C1 protein by which *NR3C1* genetic variants have been proposed to influence cortisol binding rate [4,11–13], cortisol stimulated muscle catabolism, and ultimately, muscle strength and size [15,16]. Thus, transcriptional regulation by *NR3C1* splice variants could explain why we observed associations among the muscle strength and size response to RT and the intronic SNPs *NR3C1–*2722 G>A, *NR3C1*-1887 G>A, and *NR3C1–*1017 T>C from the 5’ untranslated region, but not the missense SNP *NR3C1* +363 A>G from exon 2 for which we found no associations with the muscle strength and size response to RT.

Reasons for the sex differences we observed in the *NR3C1* genotype associations with the muscle strength and size response to RT are not clear but may be attributable to sex differences in the regulation of cortisol production. Specifically, the hypothalamic-pituitary-adrenal axis which produces cortisol is inhibited by testosterone [27] but enhanced by estrogen [28]. Men exhibit greater cortisol changes than women following progressive RT [29], but women have greater average cortisol than men [29,30]. Thus, a genetic predisposition to uptake cortisol more rapidly may influence the muscle strength and size response to RT differently between sexes, as we observed for *NR3C1–*2722 G>A, *NR3C1*-1887 G>A, and *NR3C1–*1017 T>C (Figs 1–3). Nonetheless cortisol was not measured in FAMuSS, so we can only speculate that this would be the case. However, our findings are consistent with van Rossum et al. [15] who also noted the influence of *NR3C1* genotype upon isometric and dynamic muscle strength and muscle size differed by sex.

The proportion of variance in muscle size and strength response to RT accounted for by intronic *NR3C1* SNPs was small ranging from 1.7% to 3.2%, yet statistically significant, and comparable to that of genetic variants on 17 other loci previously identified in FAMuSS [17,20–22]. Furthermore, we minimized potential unexplained variability in the muscle strength and size response to RT by enrolling a large sample of healthy, young women and men who performed standardized tests of muscle strength and size following a structured RT intervention among the various training sites. Yet, based upon our findings a large amount of the variability in the muscle size and strength response to RT remained unexplained. In addition, we did not measure important biomarkers that may have provided mechanistic insight for our findings such as cortisol nor *NR3C1* mRNAs.

In conclusion, we found the *NR3C1*–2722 G>A, *NR3C1*–1887 G>A, and *NR3C1–*1017 T>C intronic SNPs in *NR3C1* exon 1 exhibited sex dependent associations with the muscle strength and size response to RT among young, healthy European-American adults from FAMuSS. The proportion of the variability in these phenotypes explained by *NR3C1* variants was small ranging from 1.7% to 3.2% [20] consistent with previous reports that individual genetic variants explain a small portion of the variability in the muscle strength and size response to RT [17,20–22]. Further studies are therefore required to confirm our findings of the associations of 5’ untranslated region intronic *NR3C1* variants with the muscle strength and size response to RT, as well as investigate biological mechanisms that underlie the associations we found, for instance modified expression of untranslated mRNA [14].

## Supporting Information

S1 File Database. (XLS)
